# Chronic Bilateral Symmetric Anterior Shoulder Dislocation Secondary to Seizures in Chikungunya Encephalitis

**DOI:** 10.7759/cureus.32792

**Published:** 2022-12-21

**Authors:** Emerson Budhoo, Saeed R Mohammed, Dean Baiju, Ryan E Corbin, David R Deane, Paula Kassie

**Affiliations:** 1 Department of Clinical Surgical Sciences, The University of the West Indies, St. Augustine Campus, Champs Fleurs, TTO; 2 Department of Clinical Medical Sciences, The University of the West Indies, St. Augustine Campus, Champs Fleurs, TTO; 3 Department of Orthopaedic Surgery, Eric Williams Medical Sciences Complex, Champs Fleurs, TTO

**Keywords:** chikungunya encephalitis, bilateral shoulder dislocation, chikungunya, chronic shoulder dislocation, shoulder dislocation

## Abstract

Bilateral shoulder dislocations are a rare occurrence and can be categorized as either symmetric (both humeral heads dislocate in the same direction) or asymmetric (wherein the humeral heads dislocate in different directions). Shoulder dislocations may be overlooked if they are the result of systemic injury; if diagnosed >21 days after occurring, they are considered chronic dislocations.

We describe the case of a 31-year-old male who presented with an eight-week history of bilateral shoulder pain. His onset of pain coincided with a seizure secondary to Chikungunya encephalitis. Clinical and radiological examination demonstrated bilateral symmetric anterior shoulder dislocation with associated greater tuberosity fractures and extensive callus formation bilaterally. Open surgical management was performed first on the left shoulder via the deltopectoral approach. The callus was removed, the greater tuberosity fragment lifted off, reattached to the original position, and held in place with sutures and proximal humeral locking plates. The right shoulder was reduced six weeks after the left shoulder due to patient preference; the reduction utilized the same approach as with the left shoulder. Post-operatively the patient was immobilized, and physiotherapy commenced. He achieved a satisfactory range of motion four months post-operation.

Physicians should be cognizant that shoulder pain after a convulsive seizure may signify shoulder dislocation. Thorough clinical and radiological examinations are warranted in such an instance. There exists no consensus on the treatment of chronic shoulder dislocations, but it is recommended that closed reduction only be attempted up to six weeks post-dislocation due to the high risk of iatrogenic fractures and neurovascular damage beyond this time.

## Introduction

Bilateral shoulder dislocations are a rare occurrence and can be categorized as either symmetric (both humeral heads dislocate in the same direction) or asymmetric (wherein the humeral heads dislocate in different directions). Bilateral symmetric anterior shoulder dislocation (BSASD) is typically the result of trauma, but can occur due to muscular spasms [1]. When these dislocations result from a systemic injury, they may be overlooked initially, as treatment of the systemic insult is prioritized. Dislocations are defined as acute when diagnosed within 21 days of occurring and chronic when diagnosed thereafter [1].

We here report the case of a 31-year-old male who presented to a tertiary hospital with bilateral shoulder pain eight weeks after experiencing seizures secondary to chikungunya-related encephalitis and who was subsequently diagnosed with chronic BSASD.

This work is reported in accordance with the 2020 Surgical CAse REport (SCARE) criteria [2].

## Case presentation

A 31-year-old male of Afro- and Indo-Caribbean descent presented to the orthopedic outpatient clinic with an eight-week history of bilateral shoulder pain. The onset of pain in both shoulders coincided with his date of discharge from the medical ward; he had been warded for the management of seizures secondary to encephalitis from Chikungunya. He had informed the attending team of his bilateral shoulder pain and decreased range of motion (ROM) prior to discharge, but this pain was deemed to be arthralgia arising from his Chikungunya infection, and he was advised to present to the orthopedic outpatient clinic if it persisted. He attempted shoulder exercises at home but ceased due to stiffness and worsening pain.

**Figure 1:**
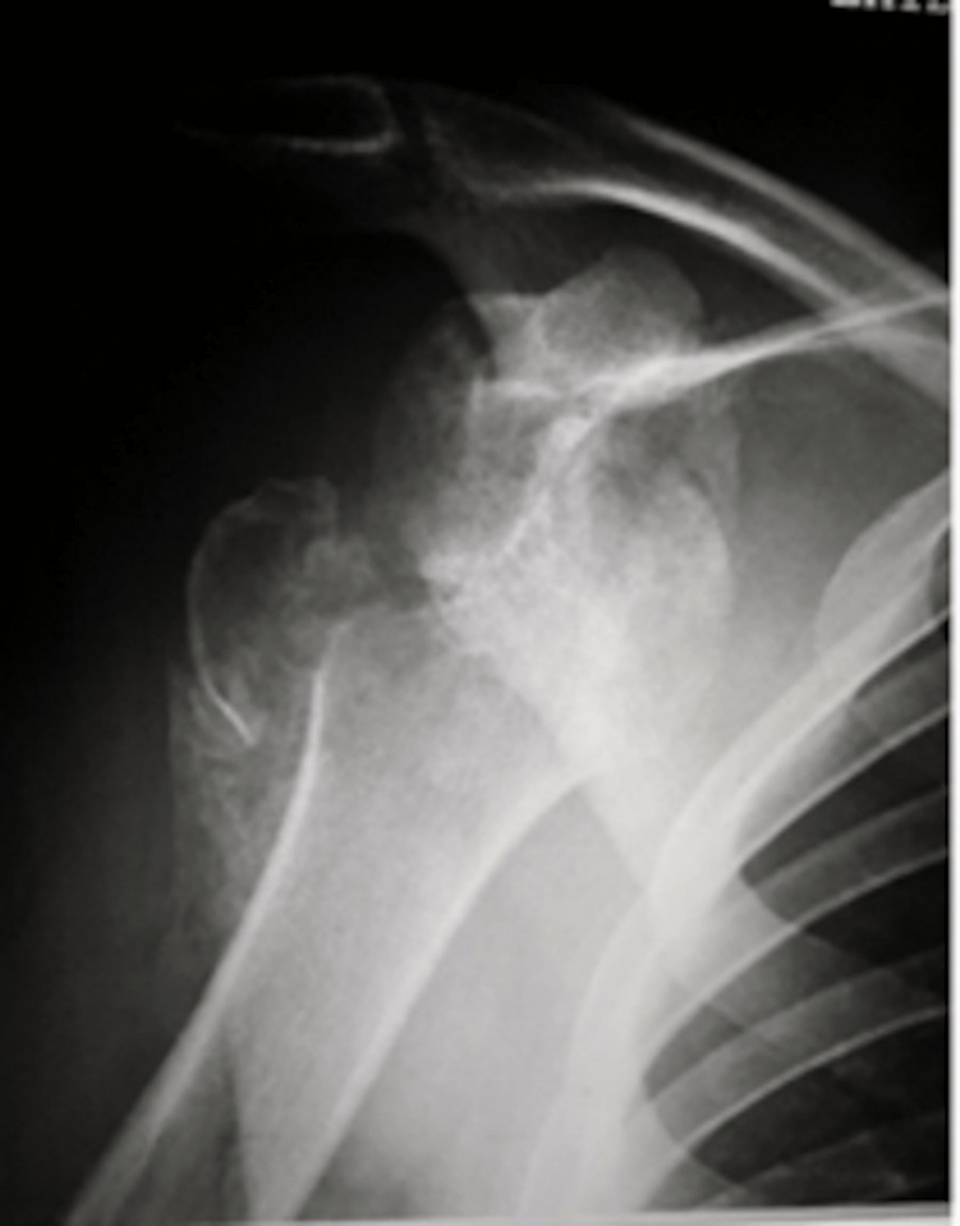
Anteroposterior view of the right shoulder at presentation

**Figure 2:**
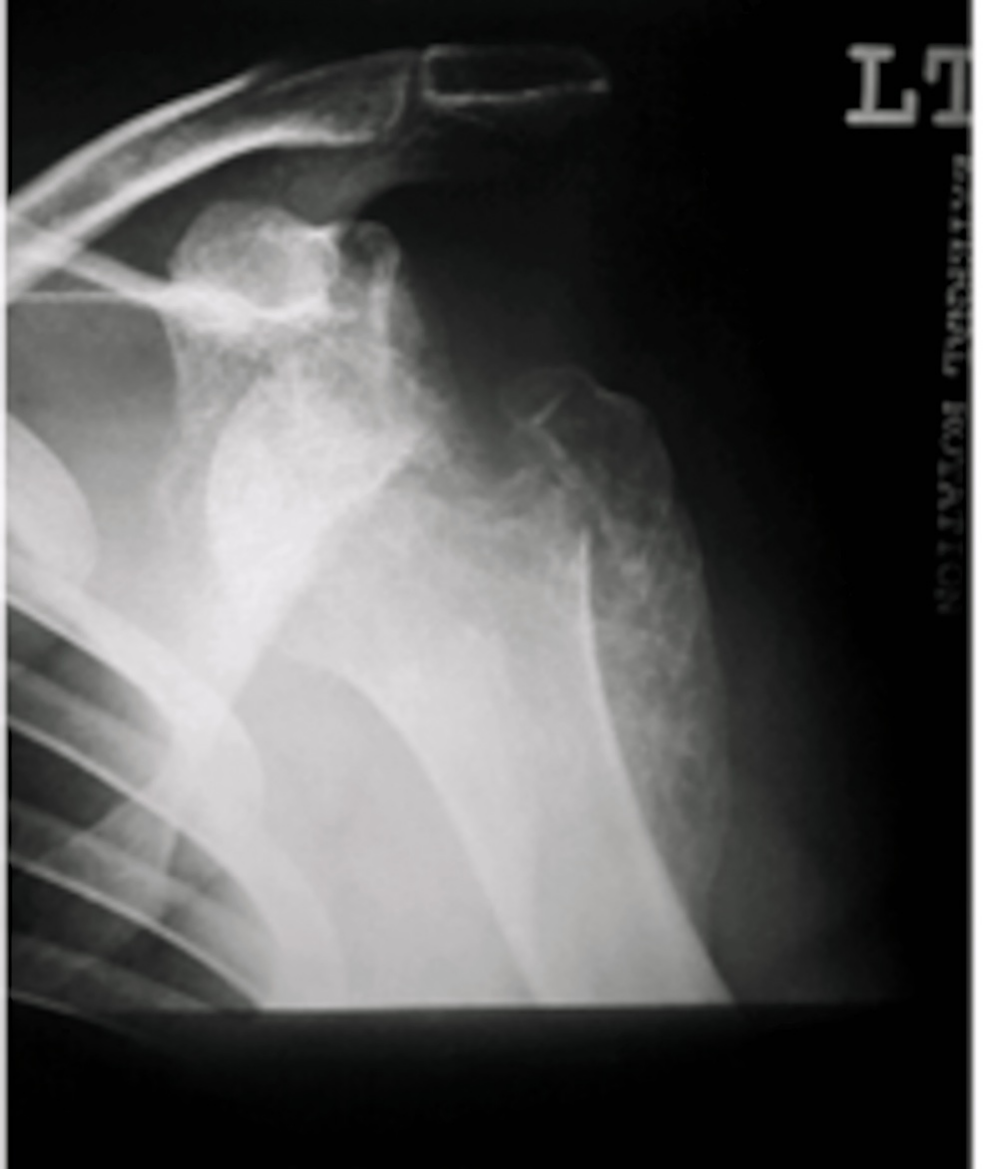
Anteroposterior view of the left shoulder at presentation

**Figure 3:**
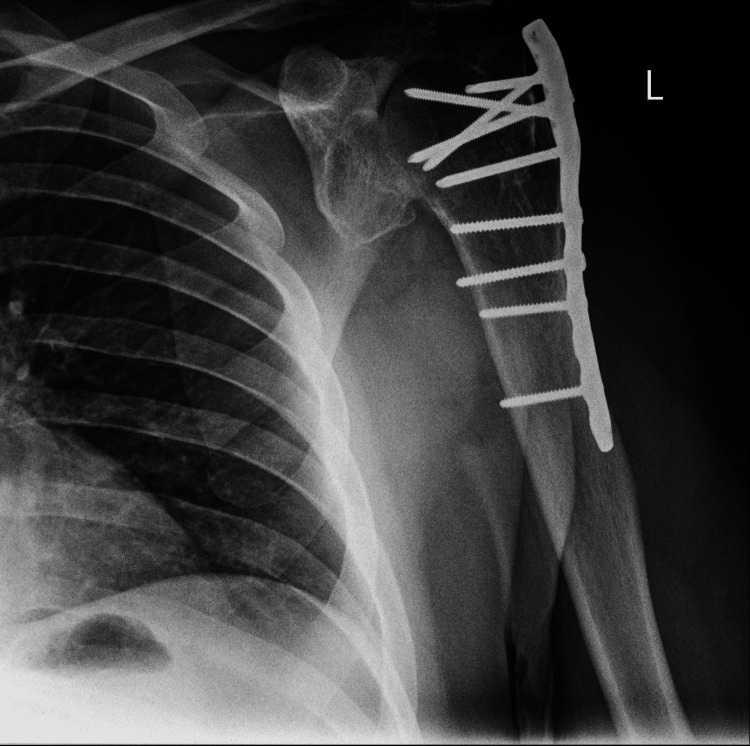
Anteroposterior view of left shoulder seven years after operation, displaying signs of avascular necrosis of the humeral head

**Figure 4:**
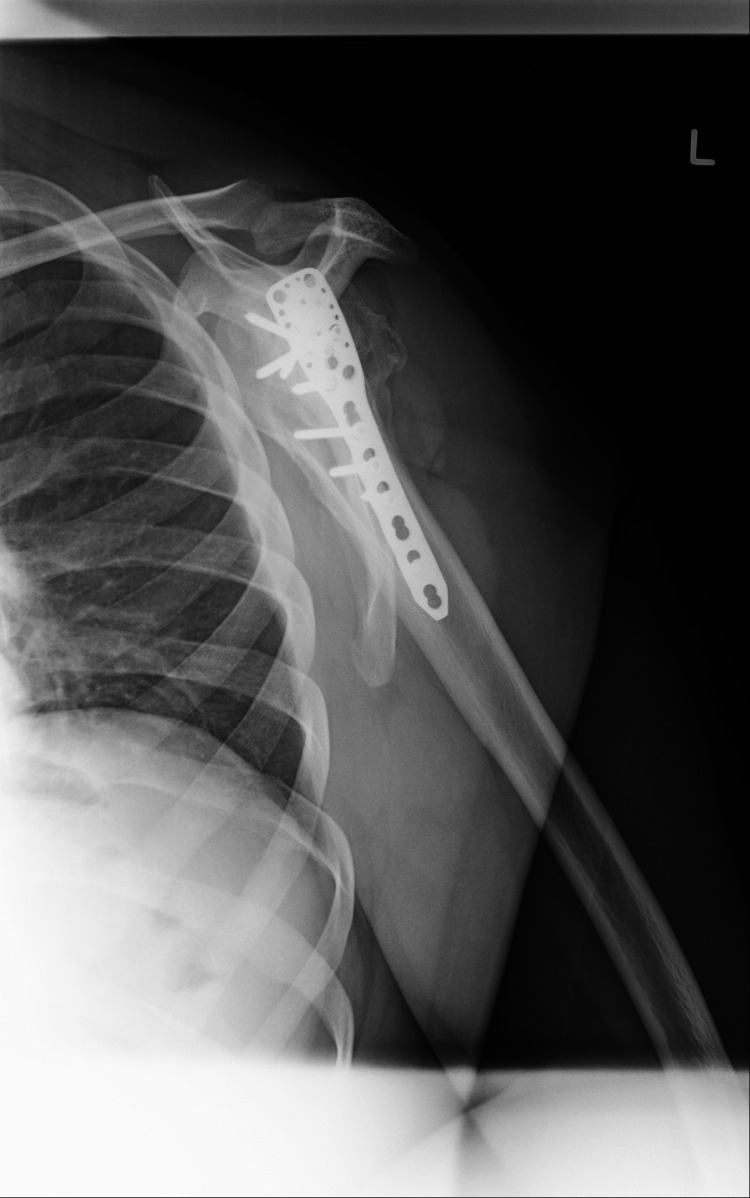
Transcapular view of left shoulder seven years after operation, displaying signs of avascular necrosis of the humeral head

**Figure 5:**
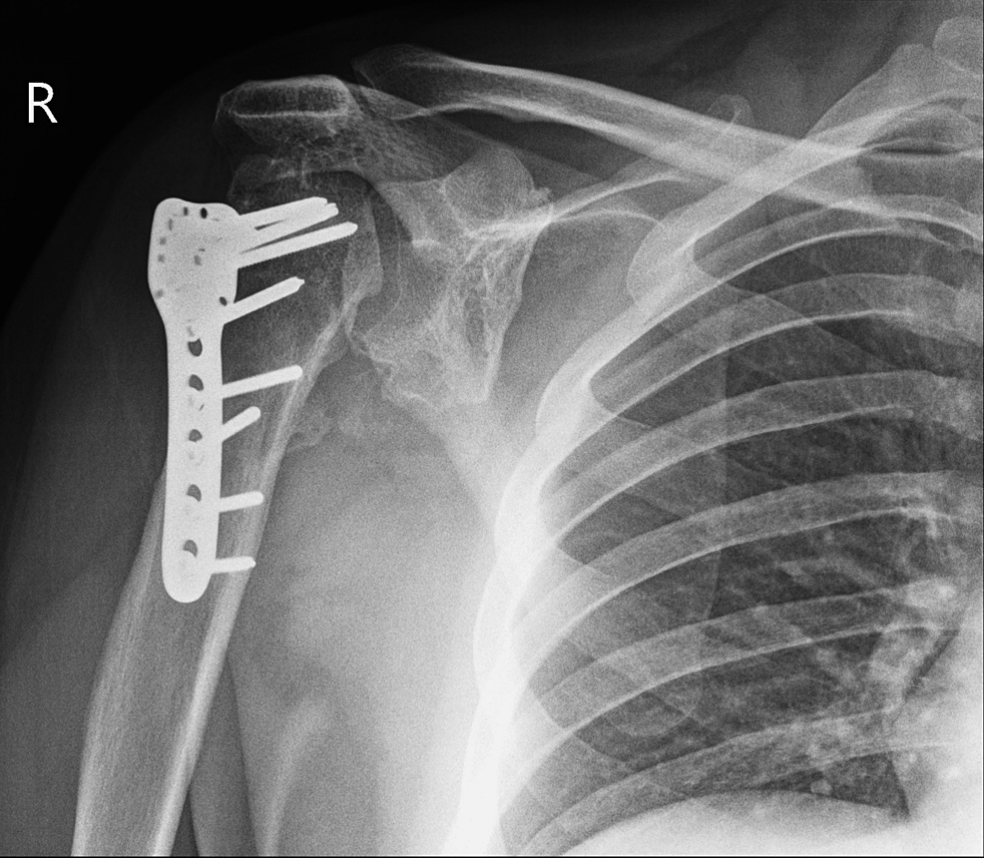
Anteroposterior view of right shoulder seven years after operation, displaying signs of avascular necrosis of the humeral head

**Figure 6:**
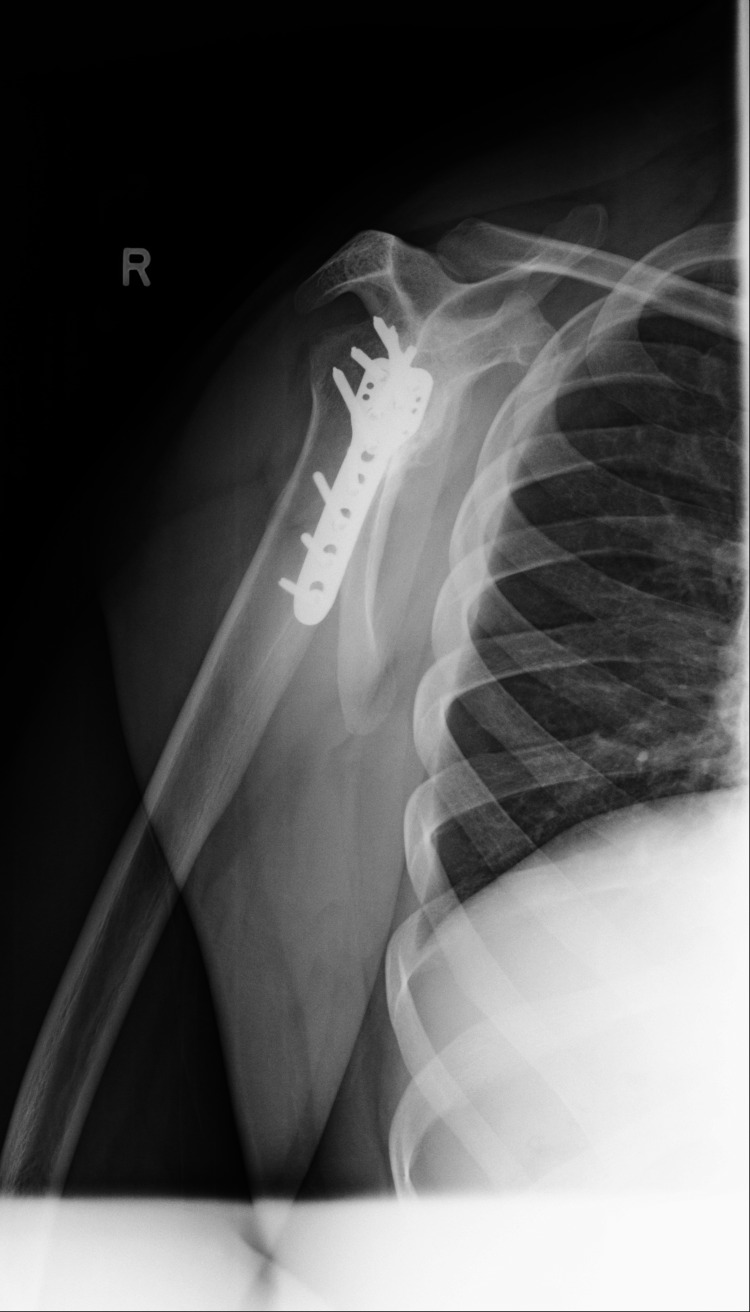
Transcapular view of right shoulder seven years after operation, displaying signs of avascular necrosis of the humeral head

Clinical examination and X-rays revealed bilateral anterior shoulder dislocation (Figures 1, 2) with associated greater tuberosity fractures bilaterally. There was extensive callus formation alongside the greater tuberosity fragment and posterolateral border of the proximal humerus. His range of motion was decreased (Table 1), whilst his neurovascular status was intact. Surgery was performed on the left shoulder five weeks after presenting to the clinic; the surgical team was led by two orthopedic surgeons with over 25 years of combined experience. Open reduction via the deltopectoral approach was utilized. The callus was removed, and the greater tuberosity fragment was lifted off and reattached to the original position and held in place with sutures and proximal humeral locking plates. A Hill-Sachs was noted and filled with an iliac crest autograft. Post-operatively the patient was placed in broad arm slings strapped to the body. Physiotherapy, consisting of isometric exercises, was commenced immediately. Passive circumduction exercises were begun two weeks post-operatively, followed by rotator cuff strengthening exercises four weeks later. The patient's range of motion and shoulder stability improved significantly, allowing independent functioning. The right shoulder was reduced six weeks after the left shoulder due to patient preference; the reduction utilized the same approach as with the left shoulder, inclusive of an iliac crest autograft. His active range of motion four months post-operation is displayed in Table 1. Seven years later, he had Constant's shoulder scores of 86 in the left shoulder (dominant hand) and 82 in the right shoulder (non-dominant hand); imaging at this time displayed signs of avascular necrosis (Figures 3-6). There were no instances of seizures after the resolution of the Chikungunya episode. The patient is currently employed in a managerial position; he reports that his shoulder function does not limit his ability to perform his professional duties.

**Table 1:** Active range of motion in the right and left shoulders at presentation and at four months post operation ROM - range of motion

Movement assessed	ROM in right shoulder		ROM in left shoulder	
	At presentation	Four months post operation	At presentation	Four months post operation
Abduction	5	65⁰	10⁰	60⁰
Flexion	10	70⁰	15⁰	65⁰
External rotation	0	60⁰	0⁰	65⁰
Internal rotation	20	60⁰	30⁰	65⁰

## Discussion

Bilateral shoulder dislocation is rarely seen, with only 57 cases reported in the literature from 1846 to 1927 [3]. Since then, numerous reports of BSASD have emerged, the majority of which occur due to traumatic injuries. Chronic BSASD is most often reported secondary to involuntary muscular contractions [1]. We conducted a literature review and identified 11 cases of chronic BSASD as a consequence of seizure activity. The characteristics of the patients reported in the literature, and the patient reported here, are presented in Table 2.

**Table 2:** Characteristics of the patients with chronic bilateral symmetric anterior shoulder dislocation as a result of seizure activity identified in the literature ROM - range of motion

Clinical Characteristic	Patient 1, reported by Yadav [4]	Patient 2, reported by Brown [5]	Patient 3, reported by Dodds and Medvecky [6]	Patient 4, reported by Lasanianos and Mouzopoulos [7]	Patient 5, reported by Abdulkadir et al. [8]	Patient 6, reported by Ahmad et al. [9]	Patient 7, reported by Mehta et al. [10]	Patient 8, reported by Raptis et al. [11]	Patient 9, reported by Chamseddine et al. [12]	Patient 10, reported by Diallo et al. [1]	Patient 11, reported by Jones et al. [13]	Patient 12, reported herein
Age, years	56	31	27	25	35	20	35	41	28	27	16	31
Sex	Male	Male	Male	Male	Male	Male	Male	Female	Male	Male	Male	Male
Cause of seizures	Convulsions upon seeing the dead body of a close relative	Epilepsy	Possibly excessive opiate use or withdrawal	Epilepsy	Tonic-colonic convulsions following a nightmare, with repeated episodes likely secondary to seizure disorder	Epilepsy	Unknown	Epilepsy	Epilepsy	Epilepsy	Epilepsy	Chikungunya encephalitis
Time elapsed from BSASD to diagnosis	<6 weeks	6 weeks	2 months	3 weeks	2 years	9 months	30 days	4 weeks	6 weeks	2 years	5.5 weeks	8 weeks
Associated fractures or neurovascular injury	Fracture-dislocation bilaterally	None	Displaced greater tuberosity fracture bilaterally	'Sizeable' greater tuberosity fracture of the left humerus and Hill-Sachs lesion bilaterally	Bilateral brachial plexus injury	None	Bilateral greater tuberosity fracture	Bilateral 'sizeable' greater tuberosity fractures. Brachial plexus neuropraxia on the right side	Coracoid tip displaced to the lateral aspect of the greater tuberosity bilaterally. Hill-Sachs lesion involving ≈20% of the humeral head present bilaterally. Tip of left coracoid process later discovered to be avulsed and completely displaced	Large Hill-Sachs lesions bilaterally	Bilateral displaced greater tuberosity fractures	Bilateral greater tuberosity fractures with extensive callus formation alongside the greater tuberosity fragment and posterolateral border of the proximal humerus
Details of management	Conservative regime consisting of assisted shoulder exercises, electrical stimulations, and infra-red therapy	Closed manipulations attempted twice, with no success. Both shoulders managed with open reduction and soft tissue repair	Open reduction of the anterior fracture dislocation and internal fixation of the greater tuberosity fracture performed; right (dominant) side first, then left 3 weeks later	Bilateral reduction with internal fixation of left greater tuberosity fracture. Broad arm slings in abduction and internal rotation thereafter, followed by physiotherapy	Patient declined open reduction surgery	Open reduction through modified Bankart procedure. Shoulders immobilized in slings for 3 weeks after procedure, then ROM exercises started	Left shoulder: closed reduction followed by 3 weeks of immobilization and intermittent physiotherapy. Right shoulder: closed reduction unsuccessful. Open reduction utilizing standard deltopectoral approach performed on second attempt. Shoulder immobilized for 3 weeks. Patient non-compliant to physiotherapy instructions, leading to partial displacement of greater tuberosity. Rehabilitation continued	Closed reduction using Kocher maneuver, followed by bilateral immobilization for 3 weeks. Physiotherapy for next 12 weeks	Closed reduction using Kocher maneuver unsuccessful on both shoulders. Open reduction using deltopectoral approach performed. Latarjet procedure performed. Post-operatively, arms immobilized in slings then rehabilitation begun 4 weeks later	Patient had successfully adapted to reduced shoulder function and surgery was thus avoided	Staged open reduction using deltopectoral approach of right then left shoulder. Coracoid osteotomy with takedown of malunited greater tuberosity fractures then Latarjet procedure performed. Post-operatively, arms immobilized for 2 weeks, followed by passion ROM of elbow for 4 weeks, then active physiotherapy for 6 weeks	Open reduction using deltopectoral approach. Greater tuberosity fragments reattached with proximal humeral locking plates. Post-operatively, arms immobilized in slings for 4 weeks, followed by physiotherapy
Clinical outcome	Reasonable functional outcome; 70⁰ of abduction bilaterally. Patient able to carry out usual activities without much difficulty	Patient regained almost full range of motion 18 months after the injury	Right (dominant) shoulder: painless ROM of 120⁰ forward flexion, 90⁰ of internal rotation, 80⁰ of abduction, 60⁰ of external rotation in abduction Left shoulder: mildly painful ROM of 100⁰ of forward flexion, 90⁰ of internal rotation, 75⁰ of abduction and 30⁰ of external rotation in abduction The patient developed avascular necrosis of the humeral heads within 6 months of surgery, but was not experiencing pain	Fully recovered ROM 4 months post-operatively	N/A	Improved ROM 5 months post-operatively	Left shoulder: Full abduction with no pain 3 months post-operatively Right shoulder: 150-160 of motion 3 months post-operatively	Right shoulder: 110⁰ forward flexion, abduction 100⁰. No parasthesias present Left shoulder: 100⁰ forward flexion and 100⁰ abduction	Painless ROM on both shoulders: 110⁰ forward flexion, 15⁰ extension, 90⁰ abduction, 20⁰ adduction, 30⁰ external rotation, 40⁰ internal rotation	Patient had Constant’s scores of 26.5 on the right and 28.5 on the left Right shoulder: 85⁰ forward flexion, 30⁰ extension, 45⁰ abduction, 45⁰ cross-body adduction, 15⁰ external rotation, 25⁰ internal rotation Left shoulder: 85⁰ forward flexion, 30⁰ extension, 85⁰ abduction, 45⁰ cross-body adduction, 15⁰ external rotation, 10⁰ internal rotation	Right shoulder: 150⁰ forward flexion, 120⁰ abduction, 30⁰ external rotation and internal rotation to mid lumbar spine Left shoulder: 110⁰ forward flexion, 90⁰ abduction, 30⁰ external rotation and internal rotation to mid lumbar spine	Right shoulder: 70⁰ forward flexion, 65⁰ abduction, 60⁰ external rotation and 60⁰ internal rotation; Left shoulder: 65⁰ forward flexion, 60⁰ abduction, 60⁰ external rotation and 65⁰ internal rotation

All patients identified in the literature were male, and 7/12 experienced seizures due to a known diagnosis of epilepsy, with one additional patient having repeated episodes, indicating a likely seizure disorder. Most cases (9/12) were associated with bilateral fractures, one case had an associated brachial plexus injury, and only 2/12 cases had no associated injury. Management typically consisted of open reduction (7/12) bilaterally, with one patient undergoing closed reduction of one shoulder and open reduction of the other after an unsuccessful attempt at closed reduction. Post-operatively, immobilization in slings followed by physiotherapy was commonly done, but few details were provided on these, and there was little homogeneity at the time of immobilization. Almost all patients (11/12) experienced reasonable functional outcomes, regardless of the approach to management; the only exception was in a case where the clinical outcome was not reported. Post-operative complications were minimal, with one patient developing avascular necrosis of the humeral heads within the follow-up period.

During a seizure, all muscles of the rotator cuff contract, but it is believed that the powerful internal rotators of the shoulder (subscapularis, pectoralis major, latissimus dorsi, and anterior fibers of the deltoid) exert more force than the relatively weak external rotators (infraspinatus, teres minor and posterior fibers of the deltoid), thus predisposing to posterior dislocation [14]. O'Connor-Read et al. [15] postulated that anterior shoulder dislocation during a seizure arises not from the seizure itself, but rather from the trauma that occurs when the shoulder contacts the ground upon falling. However, Raptis et al. [11] reported a case of chronic BSASD occurring after a patient experienced seizures without any fall or other traumatic injury. Further information regarding the position of the patient's arms during the seizure and details on any trauma occurring due to the seizure would thus allow valuable insight into the exact mechanism of BSASD resulting from seizure activity. Fracture of the greater tuberosity occurs in ≈5-30% of cases of anterior shoulder dislocation and is generally thought to be the result of either of two processes; either due to the force of the rotator cuff counteracting the anterior force on the proximal humerus, which results in avulsion of the greater tuberosity and fracture displacement as the tuberosity shears away from the humerus, or alternatively, due to impaction of the dislocating proximal humerus against the anterior glenoid rim [16].

The goal of surgical management of chronic BSASD is to reduce the dislocation bilaterally and to repair any associated fracture, soft tissue, or neurovascular injury. There exists no consensus on the treatment of chronic shoulder dislocations, but it is recommended that closed reduction only be attempted up to six weeks post-dislocation due to the high risk of iatrogenic fractures and neurovascular damage beyond this time. Recent reports indicate that open reduction of dislocations >6 weeks is associated with satisfactory outcomes [17,18]. Physicians should be aware that late surgery may increase the relative risk of avascular necrosis by as much as fivefold when compared to surgery within 48 hours [19].

We believe this to be the first reported case of chronic BSASD resulting from seizures caused by Chikungunya-related encephalitis. Chikungunya infection most commonly results in acute symptomatic infection of one to two-week duration characterized by fever, headache, rash, myalgia, and arthralgia [20]. Systemic features are atypical, with neurologic complications accounting for the majority of Chikungunya-related intensive care admissions [20]. Seizures, with or without fever, are a rare but known manifestation of Chikungunya neurologic disease [20].

This report highlights several learning points, most importantly that physicians should be cognizant of shoulder dislocation as a consequence of seizures, and thus have a high clinical suspicion for these, even in the absence of observed trauma. Physical examination, radiologic investigation, and specialist referral are essential to prevent delayed diagnosis and further complications for the patient.

## Conclusions

Bilateral shoulder dislocations are rare occurrences, but physicians should yet suspect such a diagnosis if a patient complains of shoulder pain, discomfort, or deformity following seizure activity. A thorough clinical and radiologic assessment is required to establish the diagnosis and to guide management. Late diagnosis may preclude closed reduction and necessitate surgical treatment, wherein the goal is to not only reduce dislocations but also to repair any neurovascular injury and associated fracture. We here report an unusual case of chronic bilateral symmetric anterior shoulder dislocation, associated with bilateral greater tuberosity fractures, resulting from seizures caused by Chikungunya-related encephalitis. Surgical management provided satisfactory outcomes.
